# Distinct perception profiles of gig economy employment among nursing students in Saudi Arabia: a latent profile analysis

**DOI:** 10.3389/fmed.2026.1931789

**Published:** 2026-08-31

**Authors:** Alya Alghamdi, Bandar S. Alharbi, Majed M. Aljabri

**Affiliations:** Department of Community and Psychiatric Mental Health Nursing, College of Nursing, King Saud University, Riyadh, Saudi Arabia

**Keywords:** employment models, gig economy, latent profile analysis, multinomial logistic regression, nursing students, nursing workforce, Saudi Arabia, workforce flexibility

## Abstract

**Background:**

The gig economy has become an innovative employment model with the potential to enhance workforce flexibility and support healthcare delivery. However, concerns regarding employment security, professional regulation, and continuity of care remain. Understanding how future nurses perceive this evolving employment model is important because these perceptions may influence future career decisions and the adoption of flexible workforce strategies. This study aimed to identify distinct latent profiles of nursing students based on their perceptions of gig economy employment and to examine factors associated with profile membership.

**Methods:**

We performed a cross-sectional survey among undergraduate nursing students in four public universities in Riyadh, Saudi Arabia. All eligible undergraduate nursing students who registered during the study period were invited to participate. Data were collected using a structured self-administered questionnaire. The questionnaire assessed nursing challenges, gig economy characteristics, gig economy perceptions, perceived benefits, and perceived impact. Model selection was based on information criteria, entropy, the bootstrap likelihood ratio test (BLRT), profile size, and interpretability. Multinomial logistic regression was performed to identify factors associated with latent profile membership.

**Results:**

A total of 291 nursing students were included in the analysis. Although the four-profile solution showed slightly better information criteria and higher entropy, these improvements were modest and were accompanied by increased model complexity. Considering the overall balance between statistical fit, profile size, parsimony, and substantive interpretability, the three-profile solution was selected for subsequent analyses, comprising Gig Economy Enthusiasts (39.2%), Pragmatic Evaluators (37.8%), and Personal Impact-Oriented Supporters (23.0%). Compared to Gig Economy Enthusiasts, higher perceived nursing challenges were significantly associated with increased odds of belonging to the Pragmatic Evaluators profile (OR = 2.12, 95% CI: 1.23 to 3.67). Nationality, age, and sex were not significantly associated with membership in this profile. Similarly, higher nursing challenge scores were independently associated with membership in the Personal Impact-Oriented Supporters profile (OR = 2.48, 95% CI: 1.31 to 4.69).

**Conclusion:**

Nursing students showed heterogeneous perceptions of gig economy employment, with three distinct attitudinal profiles identified. The findings highlight the importance of incorporating flexible employment concepts into nursing education and workforce planning while addressing concerns related to nursing practice.

## 1 Introduction

Healthcare systems worldwide are undergoing rapid transformation in response to population aging, increasing demand for healthcare services, workforce shortages, and advances in digital technologies (1, 2). These changes have accelerated interest in alternative workforce models that can improve workforce flexibility while maintaining the quality, accessibility, and sustainability of healthcare delivery. Among these models, the gig economy has attracted considerable attention because it enables professionals to provide services through short-term, project-based, or on-demand work arrangements that are frequently facilitated by digital platforms (3). Unlike traditional full-time employment, gig economy models emphasize flexibility in work schedules, autonomy in selecting assignments, and opportunities to engage with multiple organizations simultaneously (4). Although initially developed in sectors such as transportation and information technology, the gig economy is increasingly being explored within healthcare as a potential strategy to improve workforce responsiveness and address staffing shortages (5).

Nursing is among the healthcare professions most likely to be influenced by these evolving employment models because healthcare organizations require continuous staffing across hospitals, primary care, home healthcare, telehealth, emergency services, and community settings (6). At the same time, many healthcare organizations continue to experience shortages of qualified nurses, uneven workforce distribution, increasing workloads, burnout, and high staff turnover (7). These challenges place considerable pressure on healthcare delivery and have stimulated interest in flexible employment arrangements as a means of supplementing traditional workforce models. In the nursing context, gig economy employment refers specifically to digitally mediated, platform-enabled work arrangements in which nurses independently identify, accept, and complete short-term clinical or non-clinical assignments through online platforms or mobile applications (8). This model differs from traditional flexible employment arrangements, such as agency nursing, per diem nursing, travel nursing, temporary staffing, or independent contracting, which are typically coordinated through employers or staffing agencies and often involve predefined contractual relationships. In contrast, gig-based nursing emphasizes direct digital matching between healthcare organizations and individual nurses, greater autonomy in selecting assignments, flexible scheduling, and task-based engagement. The platforms may improve workforce responsiveness by enabling flexible scheduling, facilitating rapid matching of nursing supply with fluctuating healthcare demand, and providing nurses with greater autonomy to balance professional and personal responsibilities (6, 9). Despite these potential advantages, the implementation of gig economy employment in nursing remains controversial (8, 9). Unlike many other professions, nursing requires continuity of care, interdisciplinary collaboration, strict regulatory oversight, and clearly defined professional accountability. Consequently, flexible employment arrangements may create challenges related to continuity of care, communication within healthcare teams, employment security, income stability, career progression, and legal protection (10). Ensuring consistent clinical competency, standardized orientation, and patient safety may also become more difficult when nurses frequently work across multiple organizations. These concerns highlight the need for appropriate governance frameworks and workforce policies that balance flexibility with quality and safety (11).

These issues are particularly relevant in Saudi Arabia, where the healthcare system is undergoing extensive reform under Saudi Vision 2030 (11). The national transformation agenda seeks to strengthen healthcare quality, improve efficiency, expand digital health services, increase private sector participation, and develop a sustainable Saudi healthcare workforce. Important steps have been achieved in expanding nursing education and increasing the number of practicing nurses. Nevertheless, important workforce challenges remain, including shortages of Saudi nurses, dependence on expatriate healthcare professionals, heavy workloads, retention difficulties, and sociocultural factors that continue to influence recruitment and career development (12, 13). Addressing these challenges has become a strategic priority for achieving the objectives of the national health transformation program.

Within this context, the gig economy has been proposed as a potential workforce strategy capable of supporting several objectives of Saudi Vision 2030. Flexible employment arrangements may facilitate greater participation of Saudi nurses, improve workforce distribution across healthcare sectors, increase opportunities for women, expand telehealth services, and reduce reliance on expatriate personnel.

Although previous studies have primarily focused on practicing nurses, considerably less attention has been directed toward nursing students, who represent the future nursing workforce. Their perceptions are particularly important because attitudes formed during professional education may influence career expectations, willingness to adopt innovative employment models, and future participation in flexible healthcare delivery systems. Understanding these perceptions may therefore provide valuable evidence for nursing educators, healthcare organizations, and policymakers seeking to develop workforce strategies aligned with future healthcare needs.

Previous studies examining perceptions of gig economy employment have primarily relied on variable-centered statistical approaches, which summarize average responses across participants. Although informative, these methods assume that the study sample is relatively homogeneous and may overlook meaningful differences in individual attitudes. In contrast, latent profile analysis (LPA) is a person-centered approach that identifies unobserved subgroups of individuals with similar response patterns. By identifying distinct perception profiles rather than relying solely on average scores, latent profile analysis can provide a more nuanced understanding of nursing students’ attitudes toward gig economy employment and help identify characteristics associated with different perception patterns.

Therefore, we employed latent profile analysis to identify distinct subgroups of nursing students according to their perceptions of gig economy employment. In addition, we compared demographic characteristics and perception domains across the identified profiles and examined factors associated with profile membership using multinomial logistic regression.

## 2 Methods

### 2.1 Study design and setting

We conducted a cross-sectional study using a structured questionnaire among undergraduate nursing students enrolled in public universities in Riyadh, Saudi Arabia. All nursing students enrolled in public universities in Riyadh were invited. Data collection was conducted over a one-month period, from 18 April 2022 to 18 May 2022.

Participation was completely voluntary, and all participants provided informed consent electronically before accessing the questionnaire. To maintain confidentiality and encourage honest responses, no personally identifiable information was collected, and all responses were recorded anonymously.

### 2.2 Participants and data collection

The targeted population comprised undergraduate nursing students registered at the universities during the study period. Eligibility criteria included enrollment in a nursing program and willingness to participate in the study. Students who declined to provide consent or submitted incomplete questionnaires were excluded from the final analysis.

We used Google Forms for the self-administered electronic questionnaire. A Quick Response (QR) code linking directly to the survey was generated and distributed to students through faculty members during classroom sessions and clinical placements. Students could access the QR code from the announcement board at each university. Students scanned the QR code using their smartphones and completed the questionnaire electronically at their convenience. The online data collection approach minimized data entry errors, facilitated rapid data acquisition, and ensured secure storage of responses. A total of 328 students responded to the survey. After excluding incomplete responses, 291 nursing students (88.7%) with complete questionnaires were included in the final analysis.

### 2.3 Study instrument

Data were collected using a structured questionnaire written in English, the language of instruction for nursing education in Saudi universities. The questionnaire was developed based on previously published instruments assessing perceptions of the gig economy, nursing workforce challenges, and future employment models. Cronbach’s alpha was determined to be greater than 0.80, indicating good reliability of the questionnaire (14). The questionnaire was developed by adapting items from previously published and validated instruments that assessed concepts relevant to gig economy employment (15, 16). Minor wording modifications were made where necessary to ensure consistency with the nursing context and the objectives of the present study. No new constructs were introduced, and the conceptual domains of the original instruments were retained. Different response formats were used because the questionnaire measured conceptually distinct constructs. Most domains assessed participants’ agreement with attitudinal statements and therefore used a five-point Likert agreement scale ranging from “strongly disagree” to “strongly agree.” In contrast, the Perceived Impact domain assessed the extent to which participants considered gig economy employment advantageous and therefore employed a five-point scale ranging from “not at all advantageous” to “highly advantageous.” As latent profile analysis was performed using standardized domain scores (z-scores), differences in the original response anchors and score distributions were removed prior to model estimation.

Perceived challenges facing the nursing profession were assessed using a 16-item Nursing Challenges Scale (15, 17). The instrument was designed to evaluate students’ perceptions of current workforce barriers that may influence recruitment, retention, and future employment opportunities within the nursing profession. Specifically, the scale measures perceptions related to gender-mixed working environments, dependence on expatriate nurses, communication barriers resulting from limited cultural awareness, increased healthcare costs associated with expatriate dependency, retention of skilled nursing personnel, family concerns regarding the provision of care to patients of the opposite gender, the public image of nursing, excessive workload, work–life balance, inconvenient working schedules, sociocultural restrictions affecting Saudi nurses, lower wages, limited professional development opportunities, workplace hazards, bullying and harassment, and overall quality of life. Each item was rated on a five-point Likert scale ranging from 1 (Strongly disagree) to 5 (Strongly agree). Domain scores were calculated by averaging responses across the 16 items, with higher scores indicating greater perceived nursing workforce challenges.

The characteristics of gig economy employment were assessed using the 7-item Gig Economy Characteristics Scale, which evaluates respondents’ perceptions of the defining features of gig economy work within the nursing profession. The scale was adapted from AL-Dossary (15). The scale assesses perceptions regarding flexible work schedules, project-based employment, task diversity, technology-enabled healthcare delivery, contractual employment arrangements, freedom to choose work settings, and opportunities for generating supplementary income through flexible employment models. Each item was scored on a five-point Likert scale ranging from 1 (Strongly disagree) to 5 (Strongly agree). Higher scores indicated stronger recognition of the defining characteristics of gig economy employment.

General attitudes toward gig economy employment were evaluated using the 6-item Gig Economy Perceptions Scale, adapted from AL-Dossary (15). This scale measures participants’ overall acceptance of gig economy employment as a future workforce model in nursing. Items evaluate perceptions of flexibility in selecting assignments based on healthcare institutions, remuneration, and work schedules; the influence of employment benefits and income stability on professional motivation; the potential contribution of gig economy employment to strengthening the Saudi nursing workforce; the role of digital technologies in supporting Saudi Vision 2030; and the perceived need for legal regulations, professional standards, and governance frameworks to ensure the safe implementation of gig economy nursing practice. Responses were recorded using a five-point Likert scale ranging from 1 (Strongly disagree) to 5 (Strongly agree). Higher scores reflected more favorable perceptions of gig economy employment.

The perceived benefits of gig economy employment were assessed using the 8-item Perceived Benefits Scale, adapted from AL-Dossary (15). This instrument evaluates respondents’ perceptions of the potential advantages of gig economy employment for nurses and healthcare systems. The items address employment opportunities, reduced reliance on expatriate nurses, increased workforce participation among women and individuals with disabilities through telehealth services, recruitment flexibility, improved accessibility of nursing care, opportunities for remote work, financial independence, and professional development. All items were rated on a five-point Likert scale ranging from 1 (Strongly disagree) to 5 (Strongly agree). Domain scores were calculated as the mean of all eight items, with higher scores indicating greater perceived benefits of gig economy employment.

The anticipated influence of gig economy employment on nursing practice was assessed using the 4-item Perceived Impact Scale, adapted from AL-Dossary (15). The instrument evaluates participants’ expectations regarding the impact of gig economy employment on the quality of work life, personal development, professional development, and financial management. Unlike the previous domains, responses were recorded using a five-point advantage scale ranging from 1 (Not at all advantageous) to 5 (Highly advantageous). Higher scores indicated stronger expectations that gig economy employment would positively influence nursing practice and workforce development.

We also collected participants’ sociodemographic characteristics, including age, sex, and nationality. Age was categorized into four groups: 18 to 20 years, 21 to 23 years, 24 to 26 years, and older than 26 years. Sex was recorded as male or female, while nationality was classified as Saudi or non-Saudi.

### 2.4 Reliability assessment

The internal consistency of each study scale was assessed using Cronbach’s alpha coefficient. All five scales demonstrated good internal consistency, with Cronbach’s alpha values ranging from 0.855 to 0.900 (Supplementary Table S1). Cronbach’s alpha values were 0.856 for the Gig Economy Characteristics Scale, 0.855 for the Gig Economy Perceptions Scale, 0.900 for the Perceived Benefits Scale, 0.869 for the Perceived Impact Scale, and 0.870 for the Nursing Challenges Scale.

### 2.5 Statistical analysis

Continuous variables were summarized using means and standard deviations or medians and interquartile ranges according to their distributions, whereas categorical variables were presented as frequencies and percentages.

#### 2.5.1 Latent profile analysis

We used latent profile analysis (LPA) to identify distinct subgroups of nursing students based on their responses to the gig economy questionnaire. The latent profile analysis was conducted using the four questionnaire domains that directly assessed perceptions of gig economy employment (Gig Economy Characteristics, Gig Economy Perceptions, Perceived Benefits, and Perceived Impact). The Nursing Challenges Scale was intentionally excluded from the profile indicators because it measures perceptions of broader nursing workforce challenges rather than attitudes toward gig economy employment. The analysis included the 25 individual items representing the domains of Gig Economy Characteristics, Gig Economy Perceptions, Perceived Benefits, and Perceived Impact. Prior to model estimation, all indicator variables were standardized to ensure comparable scaling.

Latent profile analysis (LPA) was conducted in R (version 4.4.3) using the *tidyLPA* package. A series of models specifying one to five latent profiles were estimated using Gaussian finite mixture modeling, with equal variances and zero covariances between the indicators. Maximum likelihood estimation was used to estimate model parameters. Competing models were compared using the Akaike information criterion (AIC), the Bayesian information criterion (BIC), the sample size-adjusted Bayesian information criterion (SABIC), entropy, and the bootstrap likelihood ratio test (BLRT). The final model was selected by jointly considering statistical fit indices, entropy, profile sizes, model parsimony, and interpretability of the resulting profiles. The analysis was performed using the default estimation settings of the software package, including the default initialization, convergence criterion, and optimization procedures. No formal *a priori* sample size calculation for LPA was performed because there is no universally accepted sample size formula for mixture models. Instead, model adequacy was evaluated using multiple fit indices, entropy, profile sizes, and interpretability, consistent with methodological recommendations (18, 19).

#### 2.5.2 Multinomial logistic regression

To examine factors associated with latent profile membership, multinomial logistic regression was performed using the Gig Economy Enthusiasts profile as the reference category. The model included demographic variables collected in the survey (age, sex, and nationality), together with the Nursing Challenges score, which was considered a theoretically relevant contextual factor that might influence perceptions of gig economy employment. Other potentially relevant socioeconomic and educational variables, such as household income, financial strain, academic year, clinical placement experience, and prior gig work experience, were not collected and therefore could not be evaluated. The results were reported as odds ratios (ORs) with corresponding 95% confidence intervals (CIs).

All statistical analyses were performed in R version 4.4.3 (20).

## 3 Results

Table 1 presents the sociodemographic characteristics of the 291 nursing students included in the study. More than half of the participants (52.6%) were aged 21 to 23 years, followed by those aged 24 to 26 years (26.1%). Students aged 18 to 20 years accounted for 14.1% of the sample, while only 7.2% were older than 26 years. Most of the respondents were female (84.9%) and Saudi nationals (76.6%).

**Table 1:** Sociodemographic characteristics of the study participants.

Characteristic	*N* = 291 *n*(%)
Age	
18 to 20 years	41 (14.1%)
21 to 23 years	153 (52.6%)
24 to 26 years	76 (26.1%)
>26 years	21 (7.2%)
Sex	
Male	44 (15.1%)
Female	247 (84.9%)
Nationality	
Saudi	223 (76.6%)
Non-Saudi	68 (23.4%)

Internal consistency and reliability of the study scales were checked (Supplementary Table S1). All five scales demonstrated good to excellent internal consistency, with Cronbach’s alpha coefficients ranging from 0.855 to 0.900. The Perceived Benefits Scale exhibited the highest reliability (*α* = 0.900), followed by Nursing Challenges (*α* = 0.870), Perceived Impact (*α* = 0.869), Gig Economy Characteristics (*α* = 0.856), and Gig Economy Perceptions (*α* = 0.855). The findings indicate that all scales demonstrated satisfactory internal consistency and were considered reliable for subsequent latent profile and regression analyses.

Model selection yielded mixed evidence across the evaluated fit indices (Table 2). While the AIC continued to decrease and the four-profile solution produced slightly lower SABIC values and marginally higher entropy than the three-profile solution, the BIC reached its minimum for the three-profile model before increasing for subsequent solutions. Although the BLRT supported the addition of a fourth profile, the improvements in statistical fit were modest and accompanied by increased model complexity without providing substantively distinct or interpretable profiles. Furthermore, the five-profile solution was not supported by the BLRT (*p* = 0.089), indicating that it did not provide a statistically significant improvement over the four-profile model. Therefore, considering the overall pattern of fit indices, model parsimony, profile sizes, and substantive interpretability, the three-profile solution was selected as the final model. The three-profile solution demonstrated acceptable classification accuracy (entropy = 0.747), a significant BLRT (*p* = 0.010), and produced profiles that were conceptually distinct and readily interpretable. In contrast, solutions with a greater number of profiles resulted in only marginal improvements in model fit while increasing model complexity and reducing interpretability. Considering statistical fit, parsimony, and the conceptual meaningfulness of the identified profiles, the three-profile solution was selected for subsequent analyses.

**Table 2:** Model fit indices for latent profile solutions.

Number of profiles	AIC	BIC	SABIC	Entropy	BLRT *p*-value
1	20,720.5	20,904.2	20,745.6	1.000	—
2	20,581.1	20,860.3	20,619.2	0.820	0.010
3	20,462.1	20,836.8	20,513.3	0.747	0.010
4	20,380.1	20,850.3	20,444.4	0.816	0.010
5	20,377.4	20,943.1	20,454.7	0.792	0.089

The selected solution comprised 114 (39.2%) Gig Economy Enthusiasts, 110 (37.8%) Pragmatic Evaluators, and 67 (23.0%) Personal Impact-Oriented Supporters. Each profile represented a substantial proportion of the study sample. Considering the balance between statistical fit, classification quality, profile size, parsimony, and interpretability, the three-profile solution was selected for all subsequent analyses.

The sociodemographic characteristics and domain scores across the three latent profiles are summarized in Table 3. Female students constituted the majority of each profile, accounting for 83.3% of the Gig Economy Enthusiasts, 86.4% of the Pragmatic Evaluators, and 85.1% of the Personal Impact-Oriented Supporters. Across all profiles, most participants were aged 21 to 23 years (49.3 to 53.6%) and were Saudi nationals (75.5 to 79.1%).

**Table 3:** Sociodemographic characteristics and domain scores across latent profiles.

Characteristic	Gig economy enthusiasts *n* = 114	Pragmatic evaluators *n* = 110	Personal impact-oriented supporters *n* = 67
Sex			
Male	19 (16.7%)	15 (13.6%)	10 (14.9%)
Female	95 (83.3%)	95 (86.4%)	57 (85.1%)
Age (years)			
18 to 20	18 (15.8%)	13 (11.8%)	10 (14.9%)
21 to 23	61 (53.5%)	59 (53.6%)	33 (49.3%)
24 to 26	26 (22.8%)	31 (28.2%)	19 (28.4%)
>26	9 (7.9%)	7 (6.4%)	5 (7.5%)
Nationality			
Saudi	87 (76.3%)	83 (75.5%)	53 (79.1%)
Non-Saudi	27 (23.7%)	27 (24.5%)	14 (20.9%)
Nursing challenges	3.56 (3.31, 3.94)	3.63 (3.38, 3.94)	3.75 (3.31, 4.06)
Gig economy characteristics	3.86 (3.57, 4.00)	3.86 (3.57, 4.14)	3.71 (3.29, 4.00)
Gig economy perceptions	3.67 (3.33, 4.00)	3.33 (3.00, 3.50)	3.67 (3.33, 3.83)
Perceived benefits	4.25 (4.00, 4.50)	3.75 (3.50, 4.00)	3.63 (3.38, 3.88)
Perceived impact	4.25 (3.75, 4.50)	4.00 (3.50, 4.25)	4.50 (4.00, 4.75)

Values are presented as *n* (%) for categorical variables and median (Q1, Q3) for continuous variables.

Median scores for Nursing Challenges were comparable across the three profiles, ranging from 3.56 to 3.75. Likewise, perceptions of Gig Economy Characteristics showed some difference, with median scores between 3.71 and 3.86.

Greater differences were observed for Gig Economy Perceptions, Perceived Benefits, and Perceived Impact. The Gig Economy Enthusiasts profile reported the highest median score for Perceived Benefits (4.25) and a high median score for Perceived Impact (4.25). In contrast, the Pragmatic Evaluators showed the lowest median score for Gig Economy Perceptions (3.33) and lower scores for Perceived Benefits and Perceived Impact. The Personal Impact-Oriented Supporters reported the highest median score for Perceived Impact (4.50) but the lowest median score for Perceived Benefits (3.63). This means that, although they anticipated a substantial influence of the gig economy on the nursing workforce, they were less convinced about its direct benefits for individual nurses.

The identified profiles were distinguished by their response patterns across the gig economy perception domains. Demographic characteristics and Nursing Challenges were evaluated separately in subsequent multinomial logistic regression analyses to determine whether they were associated with profile membership.

**Figure 1:**
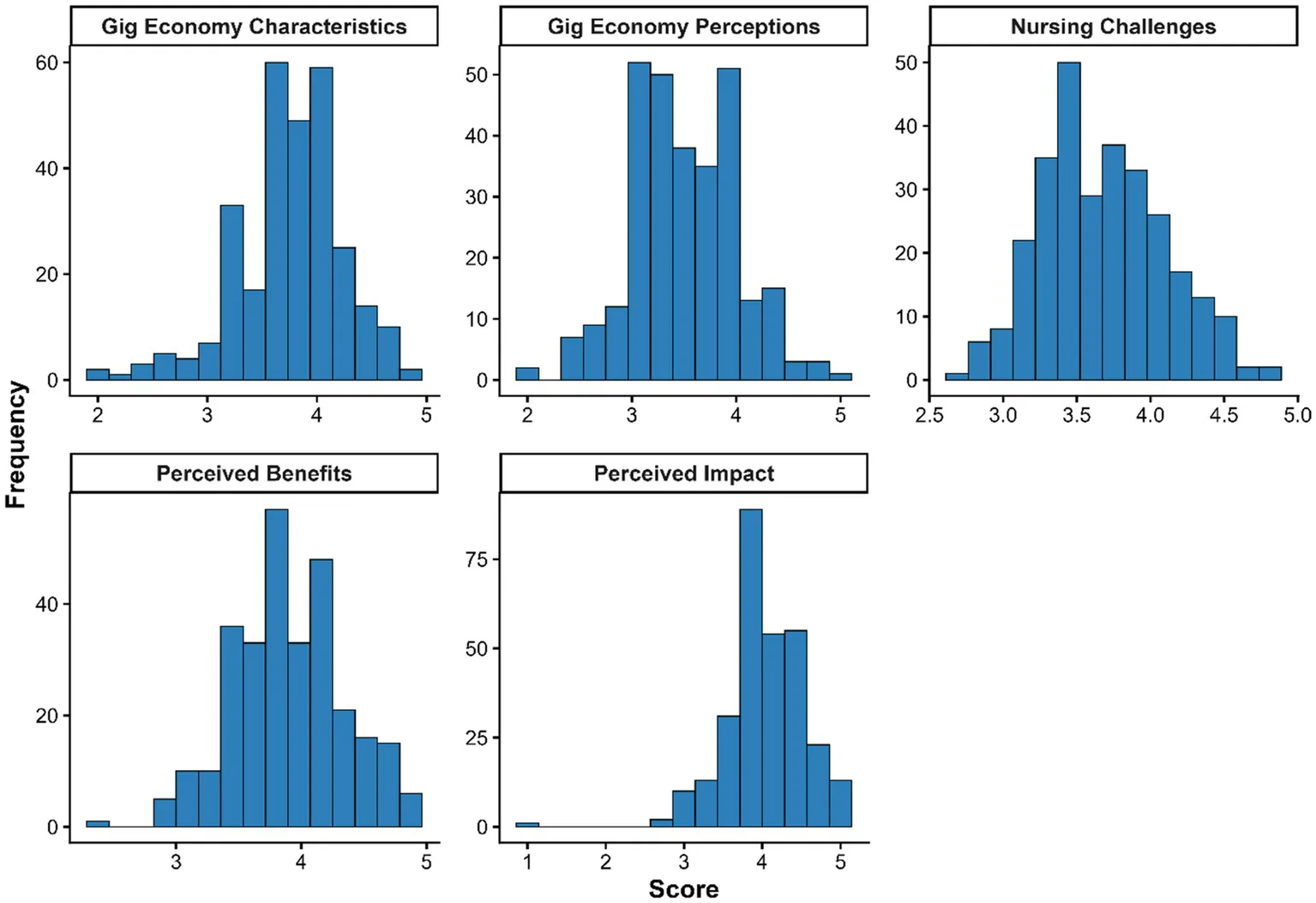
Distribution of scores across the five study domains.

The distribution of scores across the five study domains is illustrated in Figure 1. The figure shows that the distributions were concentrated above the midpoint of the five-point scale, indicating generally positive perceptions among nursing students. Perceived Impact exhibited the highest scores, with responses clustering between 4.0 and 4.5. Most participants believed the gig economy could have a substantial influence on the nursing profession. Perceived Benefits also showed a positive distribution, with most scores ranging from approximately 3.5 to 4.5. Gig Economy Characteristics demonstrated a similar pattern, with responses concentrated between 3.5 and 4.2. We found greater variability in Gig Economy Perceptions, although most responses remained above the midpoint. This suggests that, while students generally viewed gig economy employment favorably, the level of enthusiasm differed across individuals. In contrast, Nursing Challenges showed the widest spread of responses and an approximately symmetric distribution centered around 3.5 to 4.0. This means that there is heterogeneity in students’ perceptions of the existing challenges within the nursing profession.

We did not observe high extreme skewness among the five domains, although Perceived Impact and Perceived Benefits were slightly negatively skewed because a large proportion of participants reported relatively high scores. These distributions support the interpretation that nursing students generally hold favorable views toward the gig economy while differing more substantially in their perceptions of nursing challenges and overall gig economy perceptions.

**Figure 2:**
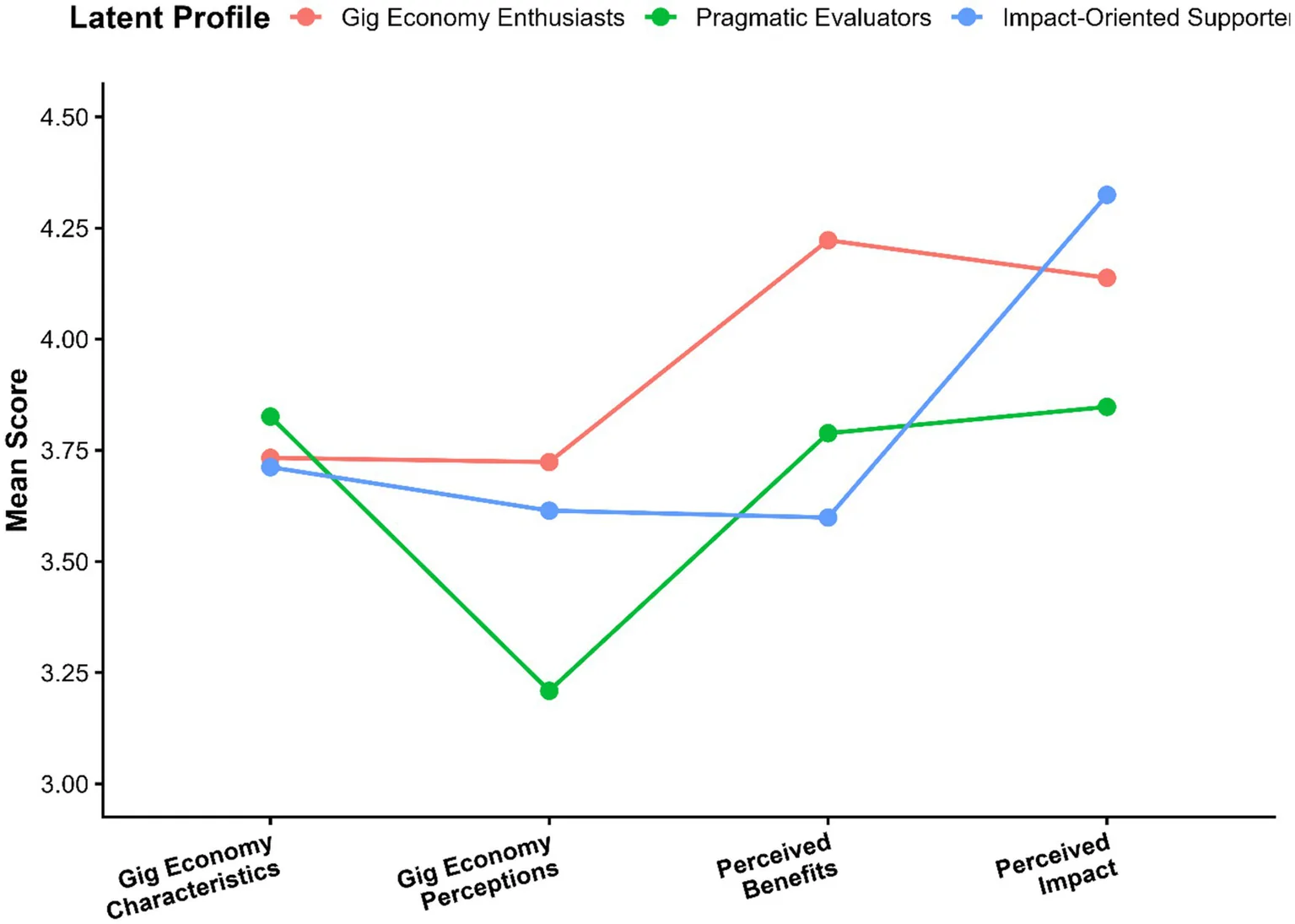
Profile-specific mean scores across the four latent profile indicator domains (Gig Economy Characteristics, Gig Economy Perceptions, Perceived Benefits, and Perceived Impact) for the selected three-profile solution.

Figure 2 illustrates the mean domain scores across the three latent profiles identified through latent profile analysis. The Gig Economy Enthusiasts profile showed consistently favorable perceptions of the gig economy, with the highest mean score for Perceived Benefits (4.22) and high scores for Perceived Impact (4.14), Gig Economy Characteristics (3.73), and Gig Economy Perceptions (3.72). This pattern indicates that students in this profile not only recognized the characteristics of gig economy employment but also viewed it as highly beneficial and impactful for the nursing profession.

In contrast, the Pragmatic Evaluators profile showed the highest score for Gig Economy Characteristics (3.83). This means that there was a strong understanding of the defining features of gig economy work. However, this profile recorded the lowest score for Gig Economy Perceptions (3.21) and relatively lower scores for Perceived Benefits (3.79) and Perceived Impact (3.85). This pattern, on the other hand, suggests a more cautious evaluation, where students acknowledged the characteristics of gig economy employment but were less convinced of its overall value and potential contribution to nursing.

The Personal Impact-Oriented Supporters profile exhibited the highest score for Perceived Impact (4.32). This indicates a strong belief that the gig economy could substantially influence the nursing workforce. However, this profile reported the lowest score for Perceived Benefits (3.60) while maintaining moderately positive perceptions of Gig Economy Characteristics (3.71) and Gig Economy Perceptions (3.61). The findings suggest that students in this profile anticipated considerable workforce implications of the gig economy but were comparatively less convinced about its direct benefits for individual nurses.

Across all three profiles, Gig Economy Characteristics showed relatively small differences (3.71 to 3.83), indicating broad agreement regarding the defining features of gig economy employment. The greatest distinctions among profiles were observed for Gig Economy Perceptions, Perceived Benefits, and Perceived Impact, demonstrating that the latent profiles were primarily differentiated by students’ evaluations of the value and potential consequences of gig economy employment rather than their understanding of its characteristics. The distinct response patterns support the validity of the three-profile solution and highlight the heterogeneity in nursing students’ attitudes toward gig economy employment.

The results of the multinomial logistic regression analysis of factors associated with latent profile membership, using Gig Economy Enthusiasts as the reference group, are summarized in Table 4. Compared to Gig Economy Enthusiasts, higher perceived nursing challenges were significantly associated with greater odds of belonging to the Pragmatic Evaluators profile (OR = 2.12, 95% CI: 1.23 to 3.67, *p* = 0.007). In contrast, nationality, age, and sex were not significantly associated with membership in this profile.

**Table 4:** Multinomial logistic regression examining factors associated with latent profile membership.

Characteristic	Odds ratio (OR)	95% CI	*p*-value
Pragmatic evaluators vs. Gig economy enthusiasts			
Age			
21 to 23	1.18	0.61, 2.29	0.620
24 to 26	0.74	0.40, 1.36	0.330
>26	0.95	0.59, 1.53	0.820
Sex			
Male	Reference	—	—
Female	1.38	0.70, 2.72	0.350
Nationality			
Saudi	Reference	—	—
Non-Saudi	1.11	0.59, 2.06	0.70
Nursing challenges	2.12	1.23, 3.67	0.007
Personal impact-oriented supporters vs. Gig economy enthusiasts			
Age			
21 to 23	1.36	0.64, 2.91	0.430
24 to 26	0.89	0.44, 1.81	0.750
>26	0.93	0.56, 1.55	0.790
Sex			
Male	Reference	—	—
Female	1.41	0.65, 3.06	0.390
Nationality			
Saudi	Reference	—	—
Non-Saudi	0.73	0.36, 1.50	0.390
Nursing challenges	2.48	1.31, 4.69	0.005

Similarly, higher nursing challenge scores were independently associated with membership in the Personal Impact-Oriented Supporters profile. Specifically, each one-unit increase in the nursing challenge score was associated with a 2.48-fold increase in the odds of belonging to this profile relative to the Gig Economy Enthusiasts profile (OR = 2.48, 95% CI: 1.31 to 4.69, *p* = 0.005). However, we did not find significant associations for age, sex, or nationality in predicting membership in the Personal Impact-Oriented Supporters profile. The findings suggest that perceptions of nursing challenges were the strongest predictor of latent profile membership, whereas demographic characteristics showed limited influence.

## 4 Discussion

### 4.1 Main findings

This study investigated nursing students’ perceptions of gig economy employment using a person-centered approach and identified distinct subgroups with different attitudinal patterns toward this emerging employment model. We identified three latent profiles: Gig Economy Enthusiasts, Pragmatic Evaluators, and Personal Impact-Oriented Supporters. The results show that nursing students do not represent a homogeneous population in their perceptions of gig economy employment. Although students generally recognized the characteristics of gig economy work, there were substantial differences in their perceptions of its benefits and anticipated impact on the nursing profession. Furthermore, multinomial logistic regression analysis showed that greater perceived nursing challenges were associated with an increased likelihood of belonging to the Pragmatic Evaluators and Personal Impact-Oriented Supporters profiles compared to the Gig Economy Enthusiasts profile. In contrast, nationality, age, and sex were not significantly associated with profile membership.

### 4.2 Comparison to previous studies

Although three latent profiles were identified, all groups generally expressed favorable perceptions of gig economy employment, with mean scores exceeding the scale midpoint across the measured domains. Thus, the profiles primarily reflect differences in the strength of positive perceptions rather than qualitatively distinct or opposing viewpoints. The most notable distinctions were observed in perceived benefits and perceived individual impacts, whereas differences in perceptions of gig economy characteristics were comparatively modest. Most previous studies examining gig economy employment in healthcare have relied on conventional statistical approaches that report average scores across the study sample (21–23). Although these studies provide valuable information regarding overall attitudes, they assume that participants respond similarly and may therefore overlook meaningful heterogeneity within the population. In this study, by applying latent profile analysis, we showed that nursing students can be grouped into distinct subpopulations with unique combinations of perceptions regarding gig economy employment. Unlike variable-centered approaches, which examine relationships among variables across the entire sample, latent profile analysis adopts a person-centered perspective by identifying subgroups of individuals with similar response patterns (24). These approaches address complementary research questions rather than representing inherently superior analytical strategies. In the present study, LPA was selected because the objective was to explore whether distinct patterns of perceptions toward gig economy employment existed among nursing students.

Our findings showed that the Gig Economy Enthusiasts represented the largest profile. They were also characterized by consistently positive perceptions of gig economy employment, particularly regarding its perceived benefits and anticipated impact. These students appeared to view flexible employment as an opportunity for greater professional autonomy, improved work–life balance, enhanced financial opportunities, and broader career development. Their favorable attitudes are consistent with previous studies reporting that flexibility is among the most attractive characteristics of gig economy employment for healthcare professionals (25). Flexible scheduling allows nurses to adjust working hours according to personal and family responsibilities while simultaneously increasing opportunities for supplemental income and professional diversification. Digital workforce platforms also facilitate rapid matching between healthcare demand and available professionals, which enables nurses to participate in multiple practice settings without long-term contractual commitments (26).

In this study, the Pragmatic Evaluators showed a distinctly different perception pattern. Although this group reported the highest recognition of gig economy characteristics, they expressed comparatively lower perceptions regarding its overall value and expected impact. This suggests that understanding the structural characteristics of gig economy employment does not necessarily translate into favorable attitudes toward its implementation. Students within this profile may recognize the operational advantages of flexible employment while simultaneously expressing concerns regarding income stability, employment benefits, career progression, continuity of care, and professional identity. Previous research has similarly reported that healthcare professionals frequently acknowledge the efficiency and flexibility of gig economy models while remaining cautious about their long-term implications for employment security and patient care (27, 28).

The Personal Impact-Oriented Supporters represented a third distinct group characterized by the highest expectations regarding the workforce impact of gig economy employment, despite reporting comparatively lower perceived personal benefits. This finding suggests that some students distinguish between benefits for individual nurses and broader benefits for the healthcare system. These students may perceive gig economy employment as an effective strategy for improving workforce flexibility, expanding healthcare access, reducing staffing shortages, and supporting national healthcare reforms while remaining uncertain about its direct advantages for their own careers. Such perspectives are particularly relevant in Saudi Arabia, where healthcare transformation under Vision 2030 emphasizes workforce modernization, digital health integration, and increased participation of Saudi healthcare professionals (11). Consequently, students may appreciate the potential system-level contributions of flexible employment models even when concerns regarding personal employment security remain.

Interestingly, we found that perceptions of nursing challenges were relatively similar across the three latent profiles, whereas high differences were observed for gig economy perceptions, perceived benefits, and anticipated impact. This pattern suggests that, although nursing students generally acknowledge common challenges facing the profession, they differ considerably in how they believe gig economy employment may address these challenges. This means that recognition of workforce problems alone might not fully explain attitudes toward flexible employment; students’ expectations regarding the effectiveness of gig economy models in addressing these challenges may also play an important role.

We found that perceived nursing challenges were significantly associated with latent profile membership. Students reporting greater nursing challenges were more likely to belong to the Pragmatic Evaluators and Personal Impact-Oriented Supporters profiles than to the Gig Economy Enthusiasts profile. This finding may initially seem counterintuitive, as one might expect students who perceive greater workforce challenges to be more supportive of alternative employment models. However, several mechanisms may explain this relationship. Students who perceive substantial challenges within nursing may develop more cautious attitudes toward emerging workforce models because they recognize the complexity of healthcare delivery and question whether flexible employment arrangements alone can adequately address issues such as staffing shortages, workload, professional recognition, and workforce retention. Rather than viewing the gig economy as a comprehensive solution, these students may consider it only one component of broader workforce reform requiring supportive policies, regulatory frameworks, and organizational investment.

The identified latent profiles highlight heterogeneity in nursing students’ perceptions of gig economy employment. These findings may provide a useful framework for future research examining whether different perception patterns are associated with educational needs, career intentions, or workforce outcomes. However, our study does not demonstrate that profile-specific educational strategies or workforce interventions are warranted, and such applications should be evaluated in future research. Although the identified profiles may be useful for informing future educational research, the present findings should not be interpreted as evidence that nursing curricula require modification. Studies evaluating students’ knowledge, educational needs, and the effectiveness of instructional interventions are needed before curriculum recommendations can be made.

### 4.3 Strengths and limitations

This study has several strengths. To the best of our knowledge, it is among the first to examine nursing students’ perceptions of gig economy employment using a person-centered analysis approach. By applying latent profile analysis, the study identified distinct subgroups of students with heterogeneous perception patterns that would have remained concealed using conventional variable-centered analyses based on mean scores. The study also employed validated measurement instruments with good internal consistency across all domains, supporting the reliability of the findings. The integration of latent profile analysis with multinomial logistic regression further strengthened the analysis by identifying factors associated with profile membership. In addition, the study addressed a timely and underexplored topic that aligns with ongoing healthcare workforce reforms and the objectives of Saudi Vision 2030, offering evidence that may inform nursing education, workforce planning, and policy development.

Several limitations should be acknowledged. We used a cross-sectional design, which precludes temporal or causal associations between perceived nursing challenges and latent profile membership. Although the study included all public universities in Riyadh, it might limit the generalizability of the findings to nursing students enrolled in private institutions or to nursing students in other countries with distinct educational systems and healthcare contexts. Although the sample size was adequate for latent profile analysis, the relatively small number of participants within some profiles may have reduced the statistical power to detect smaller associations in the multinomial regression analysis. The study relied exclusively on self-reported questionnaire data, making the findings susceptible to response bias, recall bias, and social desirability bias. Moreover, although the instruments demonstrated good internal consistency, they primarily measured perceptions and expectations rather than actual knowledge, behavioral intentions, or future participation in gig economy employment. Consequently, favorable perceptions should not be interpreted as evidence that students will ultimately pursue flexible employment models after graduation. The questionnaire was distributed electronically using a QR code, which may have introduced selection bias by favoring students who were present during data collection sessions, regularly attended classes, or were more comfortable using digital technologies. Students who were absent or less engaged may have been underrepresented. In addition, although the questionnaire completion rate was high, incomplete questionnaires were excluded from the final analysis, creating the possibility of nonresponse bias if excluded participants systematically differed from those included in the study. The Perceived Impact domain assessed the perceived degree of advantage rather than both advantages and disadvantages of gig economy employment. Consequently, the response scale may have encouraged more favorable evaluations and should be interpreted with caution. Future studies may benefit from employing balanced response options that explicitly capture both positive and negative perceptions.

The identified profiles represent statistical classifications based on the observed sample and may not be directly reproducible in different populations or settings. Alternative model specifications or larger and more diverse samples could potentially yield different profile structures. Furthermore, only selected demographic characteristics and perceived nursing challenges were examined as predictors of profile membership. Other potentially important determinants, including academic performance, socioeconomic status, previous clinical experience, career aspirations, digital literacy, personality characteristics, entrepreneurial orientation, prior exposure to flexible employment, and familiarity with telehealth technologies, were not assessed and may contribute to nursing students’ perceptions of gig economy employment.

## 5 Conclusion

Using latent profile analysis, this study identified three distinct perception profiles among nursing students based on their responses across four domains related to gig economy employment. Although all profiles generally expressed favorable perceptions, they differed in the relative strength of endorsement across the measured domains rather than representing fundamentally different viewpoints. These findings highlight heterogeneity in students’ perceptions of gig economy employment and provide a descriptive framework for understanding variation in these perceptions. However, because this cross-sectional study assessed self-reported perceptions rather than readiness, behavioral intentions, knowledge, or employment behaviors, the findings should not be interpreted as evidence of preparedness for gig employment or as support for specific educational or workforce policies.

## Data Availability

The raw data supporting the conclusions of this article will be made available by the authors, without undue reservation.
