# Supplementary material for: Distinct perception profiles of gig economy employment among nursing students in Saudi Arabia: a latent profile analysis
Source: Front Med (Lausanne). 2026 Aug 31;13:1931789. doi: 10.3389/fmed.2026.1931789 (PMC13572654; doi:10.3389/fmed.2026.1931789)
Supplement: Supplementary file 1 [file Table_1.DOCX]

**Supplementary Materials**

**Distinct Perception Profiles of Gig Economy Employment Among Nursing Students in Saudi Arabia: A Latent Profile Analysis**

Alya Alghamdi^1*^, Bandar S. Alharbi^1^, Majed M. Aljabri^1*^

^1^Community and Psychiatric Mental Health Nursing Department, College of Nursing, King Saud University, Riyadh 12375, Saudi Arabia

^*^ Shared corresponding author (Email: [aalgamdii@ksu.edu.sa](mailto:aalgamdii@ksu.edu.sa); [amajad@ksu.edu.sa](mailto:amajad@ksu.edu.sa))

| Scale | Number of Items | Cronbach's α |
| --- | --- | --- |
| Nursing Challenges | 16 | 0.870 |
| Gig Economy Characteristics | 7 | 0.856 |
| Gig Economy Perceptions | 6 | 0.855 |
| Perceived Benefits | 8 | 0.900 |
| Perceived Impact | 4 | 0.869 |

Table S1. Internal Consistency Reliability of the Study Scales
